# Shrinkage Estimation of the Realized Relationship Matrix

**DOI:** 10.1534/g3.112.004259

**Published:** 2012-11-01

**Authors:** Jeffrey B. Endelman, Jean-Luc Jannink

**Affiliations:** Robert W. Holley Center for Agriculture and Health, USDA-ARS, Cornell University, Ithaca, New York 14853

**Keywords:** realized relationship matrix, genomic selection, breeding value prediction, shrinkage estimation, GenPred, Shared Data Resources

## Abstract

The additive relationship matrix plays an important role in mixed model prediction of breeding values. For genotype matrix **X** (loci in columns), the product **XX′** is widely used as a realized relationship matrix, but the scaling of this matrix is ambiguous. Our first objective was to derive a proper scaling such that the mean diagonal element equals 1+*f*, where *f* is the inbreeding coefficient of the current population. The result is a formula involving the covariance matrix for sampling genomic loci, which must be estimated with markers. Our second objective was to investigate whether shrinkage estimation of this covariance matrix can improve the accuracy of breeding value (GEBV) predictions with low-density markers. Using an analytical formula for shrinkage intensity that is optimal with respect to mean-squared error, simulations revealed that shrinkage can significantly increase GEBV accuracy in unstructured populations, but only for phenotyped lines; there was no benefit for unphenotyped lines. The accuracy gain from shrinkage increased with heritability, but at high heritability (> 0.6) this benefit was irrelevant because phenotypic accuracy was comparable. These trends were confirmed in a commercial pig population with progeny-test-estimated breeding values. For an anonymous trait where phenotypic accuracy was 0.58, shrinkage increased the average GEBV accuracy from 0.56 to 0.62 (SE < 0.00) when using random sets of 384 markers from a 60K array. We conclude that when moderate-accuracy phenotypes and low-density markers are available for the candidates of genomic selection, shrinkage estimation of the relationship matrix can improve genetic gain.

Mixed models play an important role in the prediction of breeding values for plants and animals. Under the assumption that the breeding values are multivariate normal with genetic covariance **G**, best linear unbiased prediction (BLUP) can be used to calculate the breeding values from phenotypic data (Henderson 1984; Bernardo 2010). In the absence of molecular markers, genetic covariance can be estimated via the approximation

(1) $$\hat{G}=\text{A}\sigma_{A}^{2}$$

where each element of the numerator relationship matrix **A** is twice the coefficient of coancestry and depends on the probability of identity-by-descent (IBD) from a base population with additive genetic variance $\sigma_{A}^{2}$ (Kempthorne 1957; Lynch and Walsh 1998). We regard Equation 1 as an approximation because it depends on a number of population genetic assumptions that rarely hold in breeding populations, particularly in plant breeding (Goddard 1986; Lynch and Walsh 1998; Piepho *et al.* 2008).

When molecular markers are available, it is often assumed that the goal is to estimate the probability of IBD, but in fact, the goal is to estimate the genetic covariance, which depends on the genotypes of the causal loci and is fundamentally a state property. It follows that for a complex trait for which the infinitesimal model is a suitable approximation, **G** depends on the probability that the alleles at a random locus are identical in state, or IBS (Yang *et al.* 2010; Powell *et al.* 2010). Our first objective was to develop a theoretical framework for estimating the (realized) relationship matrix that is suitable for inbred lines and consistent with the IBS approach (*i.e.* without invoking a base population).

As the number of markers increases, the probability of IBS at a random marker approaches the probability of IBS at random genomic loci. This limiting behavior is the basis for estimators of the form (Habier *et al.* 2007)

(2) $$\hat{G}=\theta \text{XX}\prime$$

where **X** is the *n*×*m* genotype matrix for *m* markers and *n* lines, and the proportionality constant θ is fit by maximum likelihood (or REML). To see this connection more explicitly, note that when *m* bi-allelic markers are coded as {−1,0,1}, the *n*×*n* matrix of IBS coefficients (for the markers) is $\frac{1}{2}\left(m^{-1}XX\prime +J\right)$, where **J** is a matrix of ones (Piepho 2009).

Although Equation 2 is sufficient for breeding value prediction, to define a realized relationship matrix, a convention is needed concerning the scaling of the matrix. By analogy with the numerator relationship matrix, we propose that the mean of the diagonal elements equals 1+*f*, where *f* is the inbreeding coefficient of the current population

(3) $$\langle A_{ii}\rangle =1+f$$

(Beginning with Equation 3, the symbol **A** denotes the IBS relationship matrix, and the angular brackets denote the average with respect to an index, in this case, *i*.) Equation 3 requires a concept for inbreeding that is consistent with the IBS framework. Following Powell *et al.* (2010), we define the inbreeding coefficient for a single locus as the intra-individual gametic correlation, but our extension to the multi-locus case is different and emerges as an algebraic necessity during the derivation.

The strategy embodied in Equation 2, in which the IBS properties of the markers are used as a proxy for the IBS properties of any two genomic loci, requires the number of markers to be much larger than the number of lines (*m* >> *n*). However, to minimize genotyping costs in breeding programs, it is common to use low-density (*e.g.* 384) SNP arrays, in which case the number of lines may exceed the number of markers. To develop a suitable estimator for this situation, we express the realized relationship matrix in terms of the *n*×*n* variance-covariance matrix (**Σ**) for genomic loci (*i.e.* when sampling columns of the genotype matrix). Equation 2 is equivalent to estimating **Σ** with the sample covariance **S**, which in the large *m* limit is asymptotically optimal with respect to mean-squared error (MSE) (Casella and Berger 2002).

When the number of lines exceeds the number of markers, the MSE of the sample covariance matrix is no longer optimal because there are too many parameters to estimate (*n*^2^/2) relative to the number of marker data points (*nm*). This type of phenomenon is well known in the statistics literature under the name Stein’s paradox (Stein 1956; Efron 1975), and it was James and Stein (1961) who first proposed shrinkage to reduce the MSE. Yang *et al.* (2010) have proposed a shrinkage estimator for the realized relationship matrix, but it does not preserve Equation 3. We propose an alternative estimator that does not shrink the inbreeding coefficient, and we investigate its impact on the accuracy of breeding value prediction in rice, barley, maize, and pig populations.

## THEORY

### Derivation of A in terms of causal loci

Initially we work with causal loci, using an overscript tilde for variables. Eventually we will work with a marker matrix and use the same symbols without the tilde. Consider a trait with $\tilde{m}$ causal bi-allelic loci, where ${\tilde{X}}_{ik}\in \left\{0,1,2\right\}$ is the allele content at locus *k* in line *i* (the assignment of alleles is arbitrary). For an additive trait, the genetic value of line *i* can be written as

(4) $$a_{i}=\sum_{k=1}^{\tilde{m}}\left({\tilde{X}}_{ik}-2{\tilde{p}}_{k}\right)u_{k}\equiv \sum_{k}{\tilde{W}}_{ik}u_{k}$$

where the random genetic effects *u_k_* are taken to be physiological parameters of the causal loci, and $\tilde{W}$ is the centered genotype matrix. By centering the allele content of each locus (${\tilde{p}}_{k}={\left(2n\right)}^{-1}\sum_{i}{\tilde{X}}_{ik}$ are the allele frequencies), the genetic values are expressed relative to the population mean $\left(\sum_{i}a_{i}=0\right)$. Furthermore, as proved in *Appendix 1*, the additive genetic value in Equation 4 equals the breeding value, that is, twice the mean progeny value when the current population (regardless of its structure) is randomly mated.

As in the introduction, we denote the variance-covariance matrix for the breeding values by **G**

(5) $$G={\text{var}}_{u}\left[a\right]={\text{var}}_{u}\left[\tilde{W}u\right]=\tilde{W}\text{var}\left[u\right]\tilde{W}\prime =\sigma_{u}^{2}{\tilde{m}}^{-1}\tilde{W}\tilde{W}\prime$$

The subscript *u* on the variance operator indicates that it is with respect to the random genetic effects and not the genotypes—the latter are simply given and not assumed to follow any distribution. For the last step in Equation 5, we have assumed the *u_k_* are i.i.d. with constant variance $\sigma_{u}^{2}/\tilde{m}$, which is appropriate for a complex trait with many causal loci of comparable effect (*i.e.* well described by the infinitesimal model). The variance per locus is scaled by $\tilde{m}$ so that $\sigma_{u}^{2}$ is an intensive property that does not depend on the number of causal loci.

We are now in a position to decompose **G** as $A\sigma^{2}$, where **A** is the (IBS) relationship matrix satisfying Equation 3. This convention for the scaling of **A** implies that

(6) $$\text{tr}\left(\text{G}\right)=\sigma^{2}\text{tr}\left(\text{A}\right)=\sigma^{2}n\left(1+f\right)$$

where the trace operator, tr(•), sums the diagonal elements of a matrix. Genetic formulas for both the population inbreeding coefficient *f* and the variance parameter σ^2^ emerge upon applying the trace operator to Equation 5

(7) $$\text{tr}(\text{G})=\sigma_{u}^{2}{\tilde{m}}^{-1}\sum_{k=1}^{\tilde{m}}\sum_{i=1}^{n}{\left({\tilde{X}}_{ik}-2{\tilde{p}}_{k}\right)}^{2}$$

(8) $$=\sigma_{u}^{2}{\tilde{m}}^{-1}\sum_{k=1}^{\tilde{m}}\sum_{i=1}^{n}{\left[\left({\tilde{x}}_{ik1}-{\tilde{p}}_{k}\right)+\left({\tilde{x}}_{ik2}-{\tilde{p}}_{k}\right)\right]}^{2}$$

(9) $$=2\sigma_{u}^{2}n{\tilde{m}}^{-1}\sum_{k=1}^{\tilde{m}}\left({\tilde{p}}_{k}{\tilde{q}}_{k}+{\tilde{p}}_{k}{\tilde{q}}_{k}f_{k}\right)$$

Equation 7 follows from the identity $\text{tr}\left(ZZ\prime \right)=\sum_{ik}Z_{ik}^{2}$, which holds for any matrix **Z** (Searle 1971). Equation 8 follows by writing the diploid genotype as the sum of its two gametes: ${\tilde{X}}_{ik}={\tilde{x}}_{ik1}+{\tilde{x}}_{ik2}$, where ${\tilde{x}}_{ik1}$ and ${\tilde{x}}_{ik2}$ are binary variables. Equation 9, which follows from several algebraic manipulations, introduces the notation *q* = 1–*p* as well as the intra-individual gametic correlation *f_k_* at a single locus (Powell *et al.* 2010)

(10) $$f_{k}=\frac{n^{-1}\sum_{i}\left({\tilde{x}}_{ik1}-{\tilde{p}}_{k}\right)\left({\tilde{x}}_{ik2}-{\tilde{p}}_{k}\right)}{{\tilde{p}}_{k}{\tilde{q}}_{k}}$$

In *Appendix 2*, we show that *f_k_* is also the deviation from Hardy-Weinberg proportions and thus interpretable as the inbreeding coefficient for the population.

Upon comparing Equation 6 with Equation 9, we see that the coefficient of the **A** matrix is

(11) $$\sigma^{2}=2\sigma_{u}^{2}{\tilde{m}}^{-1}\sum_{k}{\tilde{p}}_{k}{\tilde{q}}_{k}=2\sigma_{u}^{2}\langle {\tilde{p}}_{k}{\tilde{q}}_{k}\rangle$$

and the inbreeding coefficient *f* is a weighted average across loci

(12) $$\begin{array}{l} f=\sum_{k}\beta_{k}f_{k}\\ \beta_{k}=\frac{{\tilde{p}}_{k}{\tilde{q}}_{k}}{\sum_{j}{\tilde{p}}_{j}{\tilde{q}}_{j}} \end{array}$$

Dividing **G** (Equation 5) by σ^2^ (Equation 11) yields the following formula for the relationship matrix

(13) $$\text{A}=\frac{\tilde{W}\tilde{W}\prime }{2\sum_{j}{\tilde{p}}_{j}{\tilde{q}}_{j}}=\frac{{\tilde{m}}^{-1}\sum_{k}{\tilde{W}}_{•k}{\tilde{W}\prime }_{•k}}{2{\tilde{m}}^{-1}\sum_{j}{\tilde{p}}_{j}{\tilde{q}}_{j}}=\frac{\langle {\tilde{W}}_{•k}{\tilde{W}\prime }_{•k}\rangle }{2\langle {\tilde{p}}_{j}{\tilde{q}}_{j}\rangle }$$

As expected, the parameter $\sigma_{u}^{2}$ cancels out and does not appear in the formula for **A**. The second step in Equation 13 is an identity from matrix algebra, in which $\tilde{W}\tilde{W}\prime$ has been written as a sum over $\tilde{m}$ matrices with dimension *n*×*n*, formed from the outer product of the columns of $\tilde{W}$ (denoted by ${\tilde{W}}_{•k}$). In the limit of the infinitesimal model, the averages in Equation 13 converge to the corresponding expected values under random sampling of genomic loci. Letting the random variables **w** and *p* denote the centered genotype and allele frequency, respectively, the result is

(14) $$\text{A}=\frac{\text{E}\left[ww\prime \right]}{2\text{E}\left[pq\right]}=\frac{\text{var}\left[w\right]+\text{E}\left[w\right]\text{E}\left[w\prime \right]}{2\text{E}\left[pq\right]}$$

### Estimating A from markers

The *n*×*m*-centered genotype matrix for the markers **W** (without a tilde) represents *m* realizations of the random variable **w** and can be used to estimate the parameters in Equation 14, the most important being the genome-wide covariance matrix **Σ =** var[**w**]. If the markers are an unbiased sample of genomic loci, then the sample covariance matrix $S=m^{-1}WW\prime -\langle W_{•k}\rangle \langle {W\prime }_{•k}\rangle$ is an unbiased estimator of the genomic parameter **Σ**. Moreover, when the number of markers is large compared to the number of lines (*m* ≫ *n*), **S** is optimal with respect to mean-squared error (Casella and Berger 2002). This leads to the following estimator for **A**

(15) $$\hat{A}=\frac{WW\prime }{2\sum_{k}p_{k}q_{k}}$$

As the number of markers decreases, the sample covariance is no longer an optimal estimator for the genome-wide covariance matrix. By shrinking the estimate, although this introduces bias, the estimation error can be decreased. One type of shrinkage estimator is the weighted average

(16) $$\hat{\Sigma }=\delta T+\left(1-\delta \right)S$$

where the shrinkage intensity *δ* ranges from 0 to 1. When *δ* = 0, there is no shrinkage and the estimator equals the sample covariance **S**. When *δ* = 1, the estimate is completely shrunk to a target **T**. The target represents a low-dimensional model that can be estimated with greater precision than **Σ** because it has fewer parameters (Schäfer and Strimmer 2005).

A common target is **T** = $\langle S_{ii}\rangle$**I** where $\langle S_{ii}\rangle$ is the mean of the diagonal elements of **S** (Ledoit and Wolf 2004). Substituting this formula into Equation 16, one can verify that the total variance is estimated without bias: $\text{E}\left[\text{tr}\left(\hat{\Sigma }\right)\right]=\text{tr}\left(\Sigma \right)$. Shrinkage is not needed for estimating the total variance because it is a single parameter. Similarly, even with as few as *m* = 96 markers, the row means of the marker matrix $\langle W_{•k}\rangle$ will be a near-optimal estimator for the genome-wide parameter E[**w**] because there are only *n* parameters to estimate from *nm* data points. Our shrinkage estimator (denoted with an asterisk) is thus

(17) $${\hat{A}}^{*}=\frac{\delta \langle S_{ii}\rangle I+\left(1-\delta \right)S+\langle W_{•k}\rangle \langle {W\prime }_{•k}\rangle }{2\langle p_{j}q_{j}\rangle }$$

To select the shrinkage intensity, we make use of results from Ledoit and Wolf (2004), who derived an analytical formula for the shrinkage intensity that minimizes the expected MSE for the covariance matrix

(18) $$\delta =\text{argmin E}\left[{\left\|\hat{\Sigma }-\Sigma \right\|}^{2}\right]$$

(The squared Frobenius norm ‖⋅‖^2^ is the sum of the squared elements of a matrix.) In *Methods*, we give the asymptotic solution to Equation 18. From this solution, a useful heuristic can be derived for when shrinkage is expected to be negligible (Ledoit and Wolf 2004)

(19) $$\delta ∼\frac{n/m}{{\left(\text{CV}\right)}^{2}}$$

where CV is the coefficient of variation of the eigenvalues of **S**. Equation 19 indicates that when the ratio of lines to markers is small compared with the dispersion of eigenvalues (*n*/*m* ≪ CV^2^), there is no need for shrinkage. This formula is revisited in *Results*.

## METHODS

### Data sets

Genotypes for several publicly available populations were used in this study:

(1) Maize diversity panel (Cook *et al.* 2012) (available at http://www.panzea.org/dynamic/derivative_data/Cook_etal_2012_SNP50K_maize282_AGPv1-111202.zip)
(2) Rice diversity panel (Zhao *et al.* 2011) (available at ftp://ftp.gramene.org/pub/gramene/CURRENT_RELEASE/data/diversity/data_download/hapmap_plink_files/div_rice34.RiceDiversity44K.hapmap.tar.gz)
(3) Commercial pig population (Cleveland *et al.* 2012) (available at http://www.g3journal.org/content/suppl/2012/04/06/2.4.429.DC1/FileS1.zip)
(4) Advanced breeding lines from the North Dakota State University 2006–2009 two-row and six-row barley breeding programs (available by querying the database at http://hordeumtoolbox.org)

For the pig population, we also used phenotypes and progeny-test-estimated breeding values (pEBV) for three anonymous traits, downloaded from the same source. Genotypes were curated by eliminating markers with more than 10% missing data and lines with more than 15% missing data. The number of lines and markers after curation are shown in Table 1. Missing marker scores were imputed with the population mean for each marker.

**Table 1:** Populations

Population	Lines (*n*)	SNPs (*m*)	*f* *^a^*	1^st^ PC*^b^*	CV*^c^*	*n*/CV^2^*^d^*
Pig	3534	52,843	0.03	0.06	4.7	1
Maize	274	44,431	0.97	0.05	1.3	1.01
2-row Barley	383	2398	0.95	0.08	2.6	0.35
2+6-row Barley	763	1884	0.97	0.32	9.0	0.06
Rice	407	31,443	0.96	0.34	7.2	0.05

a Inbreeding coefficient, estimated from the relationship matrix.

b Fraction of total variance captured by the first principal component (PC).

c Coefficient of variation (1 = 100%) for the eigenvalues of the covariance matrix.

d Quantities are relative to the pig population (= 1).

### Shrinkage intensity

Let the *n*×*m* matrix **Z** constitute *m* independent observations of an *n*-variate random variable **z** with mean **0**, for which the sample covariance matrix is **S** = *m*^−1^**ZZ**′. Ledoit and Wolf (2004) proved that the following shrinkage intensity produces an estimator that is asymptotically optimal with respect to MSE (Equation 18)

(20) $$\delta =\frac{m^{-2}\sum_{k=1}^{m}{\left\|Z_{•k}{Z\prime }_{•k}-S\right\|}^{2}}{{\left\|S-\langle S_{ii}\rangle I\right\|}^{2}}$$

For convenience, we rewrite the numerator in Equation 20 as

(21) $$\sum_{k}{\left\|Z_{•k}{Z\prime }_{•k}-S\right\|}^{2}=\sum_{k}\text{tr}\left[{\left(Z_{•k}{Z\prime }_{•k}-S\right)}^{2}\right]$$

(22) $$=\sum_{k}\text{tr}\left(Z_{•k}{Z\prime }_{•k}Z_{•k}{Z\prime }_{•k}\right)-m\text{tr}\left(S^{2}\right)$$

(23) $$=\sum_{k}\sum_{ij}Z_{ik}^{2}Z_{jk}^{2}-m\text{tr}\left(S^{2}\right)$$

(24) $$=m\sum_{ij}\left(\Gamma_{ij}-S_{ij}^{2}\right)$$

Equations 22 and 23 follow from cyclic permutation properties of the trace and the definition of **S**. Equation 24 introduces the matrix

(25) $$\Gamma =m^{-1}\left[Z_{\mathrm{ik}}^{2}\right]\left[Z_{\mathrm{ik}}^{2}\right]\prime$$

where $\left[Z_{ik}^{2}\right]$ is the matrix formed by squaring the elements of **Z**. In practice, the shrinkage intensity is confined to the interval [0,1] when Equation 20 yields a value outside this range (Ledoit and Wolf 2004). When applying these formulas to the genomic data, for **Z** we used the centered genotype matrix **W** adjusted to have zero row means: $Z_{ik}=W_{ik}-m^{-1}\sum_{k}W_{ik}$.

This shrinkage algorithm has been implemented as part of the rrBLUP package for R, version 4.0 (Endelman 2011; R Development Core Team 2011).

### Simulation and analysis

Simulated traits were constructed by first generating additive genetic values from the multivariate normal distribution, with variance equal to the full-marker relationship matrix (hence, σ^2^ = 1). Independent normal deviates with variance $\sigma_{e}^{2}$ were added to generate phenotypes, and the $\sigma_{e}^{2}$ parameter was modulated to simulate traits with different phenotypic accuracies. Figure 3 was generated with $\sigma_{e}^{2}$ = 3 for the plant species and $\sigma_{e}^{2}$ = 2 for the pigs. Figure 4 is based on 10,000 simulations, with log_2_$\sigma_{e}^{2}$ chosen from a uniform(−1,7) distribution, and results were binned by realized phenotypic accuracy in 0.1 increments.

Mixed model prediction of breeding values was conducted with the model

(26) y=μ1+a+ε

where **y** is the vector of phenotypes, *μ* is a fixed effect, **1** is a column vector of ones, $a∼N\left(0,\hat{A}\sigma^{2}\right)$ is the vector of breeding values with estimated relationship matrix $\hat{A}$, and the residuals are $\varepsilon ∼N\left(0,I\sigma_{e}^{2}\right)$. Computations were done with R package rrBLUP (Endelman 2011), which estimates variance components by REML using the eigenvalue decomposition algorithm of Kang *et al.* (2008). With this algorithm, the inverse phenotypic covariance matrix ${\hat{V}}^{-1}$ is readily generated, after which the BLUE and BLUP solutions for the fixed and random effects, respectively, can be calculated using standard formulas (Searle *et al.* 1992)

$$\begin{array}{l} \hat{\mu }={\left(1\prime {\hat{V}}^{-1}1\right)}^{-1}1\prime {\hat{V}}^{-1}y\\ \hat{a}={\hat{\sigma }}^{2}\hat{A}{\hat{V}}^{-1}\left(y-\hat{\mu }1\right) \end{array}$$

Accuracy was defined as the Pearson correlation coefficient between the genomic estimated breeding values (GEBV = $\hat{a}$) and either the true breeding values (in the simulation) or the progeny-test-estimated breeding values (pEBV) for the pig traits.

## RESULTS

Table 1 lists several attributes of the five populations used in this study. The population sizes ranged from *n* = 274 (maize) to *n* = 3534 (pig). The pig, maize, and rice populations had 30–50K SNPs, whereas only 2K SNPs were available for the barley populations. The inbreeding coefficient (*f*) for each of the four plant populations, calculated from the mean diagonal element of the relationship matrix, was near 1 as expected for inbred lines (imputing missing markers with the population mean introduced low levels of heterozygosity). The pig population was outbred with *f* = 0.03.

Both structured and unstructured populations were included. The rice population was a diverse panel of several distinct types (*indica*, *japonica*, *Aus*, *etc*.) identifiable with principal component (PC) analysis (Zhao *et al.* 2011). The observation that 34% of the total variation was captured by the first PC indicates its highly structured nature (1^st^ PC in Table 1). We intentionally grouped the 2-row and 6-row barley lines, which as separate populations are unstructured (1^st^ PC < 10%) and derived from different breeding programs, into one population to create a second structured population for analysis (32% explained by 1^st^ PC). The pig and maize populations were relatively unstructured (1^st^ PC < 10%).

**Figure 1:**
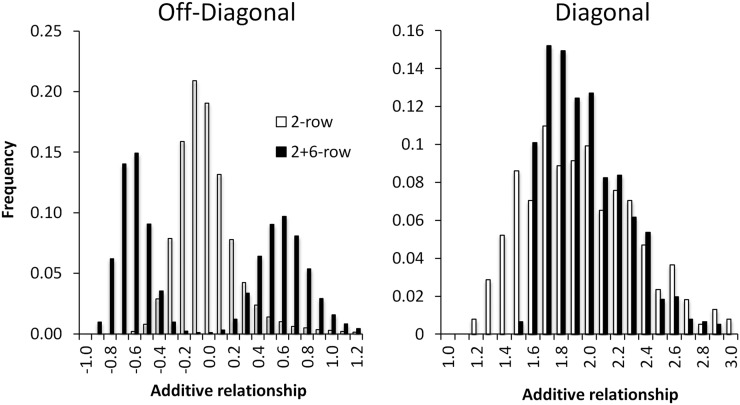
Histograms of entries in the realized relationship matrix for the 2-row and 2+6-row barley populations. The diagonal elements have a mean of 1 + *f* ≈ 2 for inbred lines, while the off-diagonal elements have a mean of −(1 + *f*)/*n* ≈ 0. The bimodal distribution of the off-diagonal elements reveals the highly structured nature of the 2+6-row barley population. The positive peak contains relationships between lines with the same row number, while the negative peak is between lines with different row numbers.

Population structure can also be detected from a histogram of the realized relationship coefficients. Figure 1 contrasts the unstructured 2-row barley population with the structured 2+6-row population. Because the relationship coefficients are expressed relative to the current population, the mean of the off-diagonal elements (left panel) is −(1 + *f*)/*n*, which is essentially 0 for populations with hundreds of lines or more. Despite having the same mean, the histogram for the 2-row population is unimodal, whereas that for the 2+6-row population is bimodal. The positive peak in the bimodal distribution arises from relationships between lines with the same row number, while the negative peak corresponds to relationships between lines with different row numbers. The highly structured rice population also has a diffuse distribution of off-diagonal elements, whereas the pig and maize distributions are unimodal (supporting information, Figure S1).

The right panel in Figure 1 shows the distribution of diagonal elements in the realized relationship matrices for the 2-row and 2+6-row barley populations. Although the mean of the diagonal elements is 1+*f* and thus at most 2, the individual coefficients can be larger than 2, unlike the diagonal elements of the numerator relationship matrix. The interpretation of the diagonal coefficients in terms of inbreeding is discussed below.

### Shrinkage to minimize MSE

**Figure 2:**
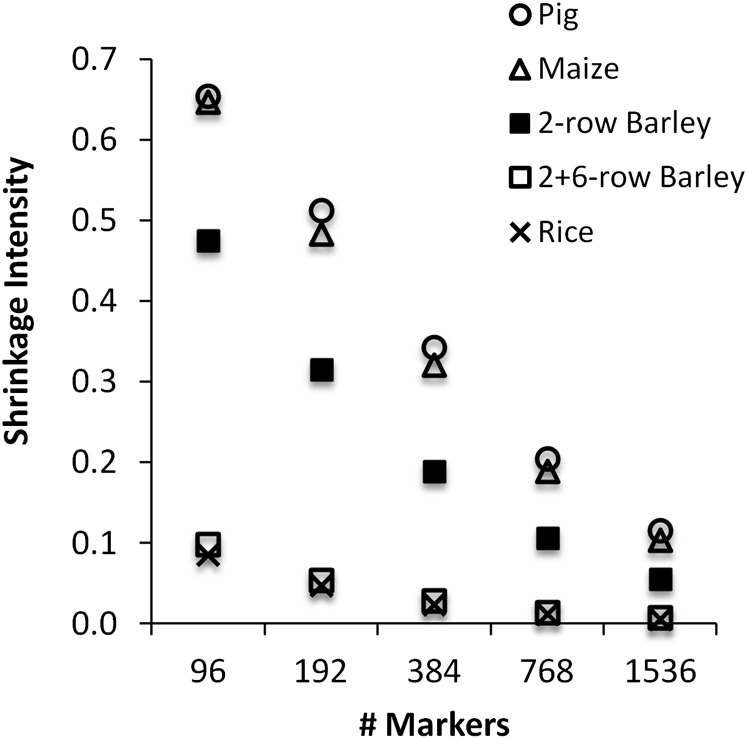
Shrinkage intensity to minimize the expected MSE. Each point is the mean from 20 random subsets of markers (SE < 0.01). As expected, the optimal shrinkage decreased as the number of markers increased. There was little shrinkage for the structured populations (rice, 2+6-row barley) because of their high eigenvalue dispersion (see CV in Table 1).

For each of the five populations, relationship matrices were estimated from random subsets of markers, with the shrinkage intensity chosen to minimize the expected MSE. As shown in Figure 2, for every population, the shrinkage intensity approached zero as marker number increased, but there were clear differences in the amount of shrinkage at low marker density. With 384 markers, the two structured populations (rice and 2+6-barley) had less than 3% shrinkage compared with nearly 20% shrinkage for the 2-row barley and over 30% shrinkage for the maize and pig populations.

These trends can be understood in terms of the heuristic in Equation 19, in which (for a given marker density) the shrinkage intensity depends on the ratio *n*/CV^2^ between population size (*n*) and the coefficient of variation (CV) for the eigenvalues of the *n×n* covariance matrix. Because the leading principal components in a structured population account for a large amount of the total variation, such populations have high eigenvalue CV. As shown in Table 1, the rice and 2+6-row barley populations had the highest CV values (7.2 and 9.0, respectively), while the maize population had the lowest at 1.3. The final column in Table 1 shows the ratio *n*/CV^2^ relative to the pig population (= 1). Although the pig population was nearly 13 times the size of the maize population, its CV was 3.6 times larger, leading to nearly identical *n*/CV^2^ ratios and shrinkage intensities in Figure 2. The two structured populations had the smallest *n*/CV^2^ ratios and thus also the least shrinkage in Figure 2. The 2-row barley population was intermediate between these extremes.

**Figure 3:**
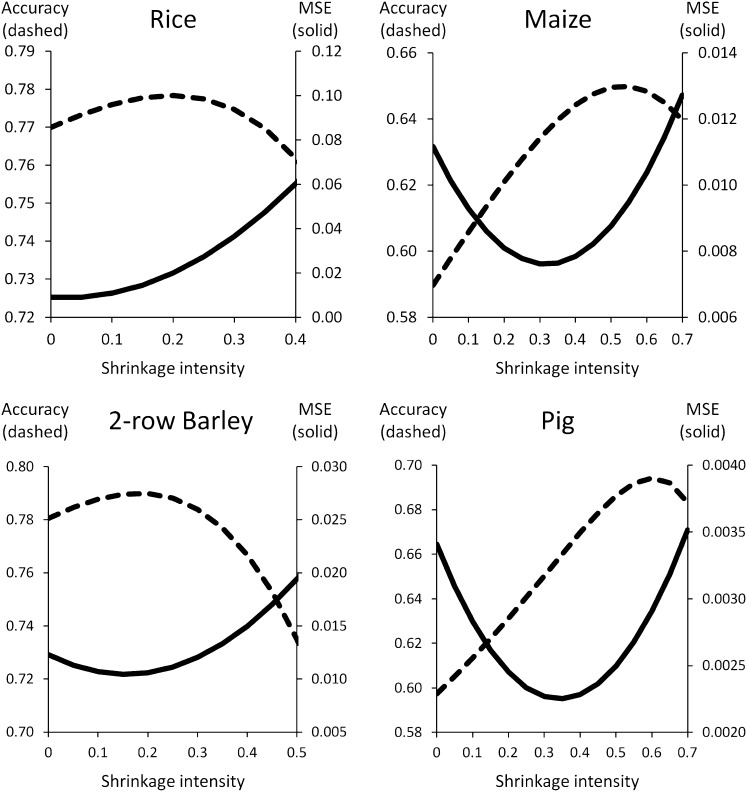
Maximizing accuracy *vs.* minimizing MSE. At shrinkage intensities ranging from 0 to 0.7, with 0.05 increments, the relationship matrix was calculated for random sets of 384 markers. In each replicate, the MSE was calculated relative to the full marker relationship matrix (MSE = *n*^−2^‖**A**_384_ − **A**_full_‖^2^), and GEBV accuracy was estimated using simulated phenotypes. The two curves (dashed = accuracy, solid = MSE) show the mean from 40 simulations (SE less than 3% of the mean).

The shrinkage intensities in Figure 2 were based on minimizing the *expected* MSE, as determined from a reduced marker set. Figure 3 (using *m* = 384 markers) shows that this approach did in fact minimize the *actual* MSE between the full-marker relationship matrix and that based on the reduced marker set (see Figure S2 for 2+6-row barley). The solid lines show the MSE as a function of the shrinkage intensity (in 0.05 increments), and in every case, the minimum was attained near the value indicated in Figure 2. For the rice and 2+6-row barley populations, the minimum MSE was attained at 5% shrinkage *vs.* 2–3% shrinkage based on the expected MSE. The correspondence was equally good for the unstructured populations: 2-row barley = 15% actual *vs.* 19% expected; pig = 35% actual *vs.* 34% expected; maize = 30% actual *vs.* 32% expected.

### Maximizing accuracy

Minimizing the MSE, although theoretically tractable, is not in itself particularly useful. A more meaningful criterion is maximizing the accuracy of breeding value prediction. The dashed curves in Figure 3 show the effect of shrinkage on prediction accuracy, as measured by the correlation between GEBV (using the shrunken relationship matrix and all phenotypes for training) and true breeding values simulated with the full marker matrix. The results indicate that shrinkage based on minimizing MSE is somewhat conservative with respect to maximizing accuracy. This follows from the observation that the maximum in the accuracy curve occurred at higher shrinkage than where MSE was minimized. For the maize, rice, and pig populations, the shrinkage intensity needed to minimize MSE was 0.20–0.25 less than for maximizing accuracy. This difference was somewhat smaller for the barley populations, but they only had 2K markers for estimating the full marker relationship matrix.

**Figure 4:**
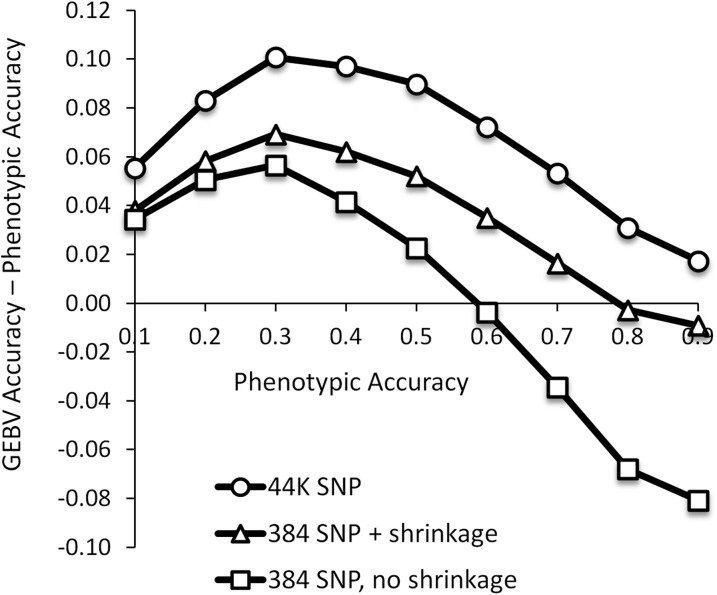
Prediction accuracy for simulated phenotypes in the maize population. The three curves show the difference between GEBV accuracy and phenotypic accuracy as a function of phenotypic accuracy (SE < 0.004 not shown). GEBV accuracy was highest using all markers, followed by 384 SNPs with shrinkage. All three prediction methods peaked when phenotypic accuracy was 0.3, while the accuracy gain due to shrinkage increased monotonically with phenotypic accuracy. Phenotypic accuracies between 0.4 and 0.6 represented a “sweet spot” for shrinkage: in this range, heritability was high enough for shrinkage to substantially improve GEBV accuracy but not so high that phenotypes were more accurate.

Figure 4 compares GEBV accuracy against phenotypic accuracy in the maize population for a range of simulated heritabilities. The three curves correspond to (1) using all 44K markers, (2) using a random set of 384 SNPs with shrinkage, and (3) using 384 SNPs without shrinkage. For all three methods, the maximum GEBV accuracy relative to phenotypic accuracy was observed at a phenotypic accuracy of 0.3 (SE < 0.004). Comparing the two lower curves, one sees that shrinkage improved GEBV accuracy with 384 markers, and the accuracy gain increased with heritability. At a phenotypic accuracy of 0.9, shrinkage improved GEBV accuracy by 0.07 on average.

Figure 4 also illustrates that phenotypic accuracy can be superior to GEBV accuracy for highly heritable phenotypes. When phenotypic accuracy was above 0.6, it surpassed GEBV accuracy using random sets of 384 SNPs without shrinkage, and the crossover with shrinkage occurred at phenotypic accuracy equal to 0.8. This phenomenon arises because low-density markers sample the genome incompletely, leading to discrepancy between the true and estimated relationship matrices. If the sampling error is large enough, the accuracy of the phenotypes is corrupted rather than improved through the mixed model analysis. The “sweet spot” for shrinkage in this simulation was at phenotypic accuracies between 0.4 and 0.6. In this range, GEBV accuracy was substantially improved by shrinkage and was also higher than phenotypic accuracy.

These trends were confirmed by our analysis of three anonymous traits in the pig population, for which progeny-test-estimated breeding values (pEBV) are available to calculate accuracy (Cleveland *et al.* 2012). Table 2 compares the accuracy of phenotypes, high-density SNPs (53K), and low-density SNPs (random sets of 384), both with and without shrinkage. The top row for each trait shows the accuracy for individuals with measured phenotypes; the bottom row is for individuals without a measured phenotype. Looking at the last two columns, one sees a clear benefit to using shrinkage for predicting the breeding value of phenotyped individuals, and this benefit increased with heritability. For trait T3 (*h*^2^ = 0.38), shrinkage increased 384 SNP GEBV accuracy from 0.56 to 0.62, a gain of 0.06 (*P* < 10^−10^ by paired *t*-test). For traits T4 and T5 (*h*^2^ ≈ 0.6), the accuracy gain from shrinkage was 0.09 and 0.10, respectively, but phenotypic accuracy was still higher. With a phenotypic accuracy of 0.58, trait T3 appears to be in the sweet spot: GEBV accuracy was improved by shrinkage and was also higher than phenotypic accuracy.

**Table 2:** Prediction accuracies for pig traits

Trait	*h^2^**^a^*	*n*	Phenotypic Accuracy*^b^*	GEBV*^c^* Accuracy 53K SNP	GEBV Accuracy 384 SNP + Shrinkage	GEBV Accuracy 384 SNP, No Shrinkage
T3	0.38	3141*^d^*	0.580	0.690	0.617 (0.002)*^e^*	0.561 (0.002)
		393	–	0.465	0.370 (0.007)	0.370 (0.007)
T4	0.58	3152	0.751	0.809	0.718 (0.002)	0.630 (0.002)
		382	–	0.569	0.469 (0.004)	0.469 (0.004)
T5	0.62	3184	0.734	0.765	0.678 (0.003)	0.584 (0.003)
		350	–	0.520	0.429 (0.012)	0.429 (0.012)

a Heritability reported by Cleveland *et al.* (2012).

b Accuracy = correlation with progeny-test-estimated breeding values.

c Genomic-estimated breeding values (GEBV) calculated using all phenotyped individuals.

d Within each trait, the top row is for individuals with a measured phenotype; the bottom row is for individuals without a phenotype.

e Mean and SE based on 20 random sets of 384 markers.

Table 2 shows that shrinkage did not improve GEBV accuracy for the unphenotyped pigs, nor have we observed any benefit in simulations. For example, even with as few as 96 markers, where the gains in GEBV accuracy were 0.1–0.2 in the maize population when training on all phenotypes, there was no accuracy gain when predicting unphenotyped lines.

## DISCUSSION

Although we have built upon the work of Powell *et al.* (2010) and Yang *et al.* (2010), our results are different from their unified additive relationship, or UAR, model. The UAR model assumed a genetic model with standardized coefficients for the causal loci

(27) $$\begin{array}{l} a_{i}=\sum_{k}{\tilde{Z}}_{ik}u_{k}\\ {\tilde{Z}}_{ik}=\frac{{\tilde{X}}_{ik}-2{\tilde{p}}_{k}}{\sqrt{2{\tilde{p}}_{k}{\tilde{q}}_{k}}} \end{array}$$

Equation 27 has the undesirable property that the genetic values of lines possessing a rare causal allele tend to infinity as the allele frequency approaches zero. The marker-based estimate of the off-diagonal elements in the UAR matrix is

(28) $$UAR_{ij}=m^{-1}\sum_{k=1}^{m}\frac{\left(X_{ik}-2p_{k}\right)\left(X_{jk}-2p_{k}\right)}{2p_{k}q_{k}}$$

which also tends to infinity for lines possessing a rare marker allele as its frequency approaches zero. Such divergent behavior does not occur in the estimators we have derived. Our formula for high-density markers (Equation 15) is identical to the first formula proposed by VanRaden (2008) for use with an unselected, outbred base population. Our IBS derivation provides rigorous justification for using this formula in any population when the number of markers is much larger than the number of lines.

In the numerator relationship matrix, each diagonal element equals one plus the probability that the two alleles at a randomly chosen locus are IBD from the base population. As this probability lies between 0 and 1, the diagonal elements in the numerator relationship matrix range from 1 to 2. It was evident from Figure 1 that the diagonal elements in the realized relationship matrix can fall outside this range. In the UAR model, the diagonal elements have been modified to lie in the range 0–2 (Yang *et al.* 2010; Powell *et al.* 2010), but this has the effect of creating an improper covariance matrix for the breeding values (*i.e.* it may no longer be positive semidefinite).

From the formula for the realized relationship matrix in Equation 13, the analog to the inbreeding coefficient for an individual is

(29) $$\phi_{i}=\frac{\sum_{k}{\left({\tilde{X}}_{ik}-2{\tilde{p}}_{k}\right)}^{2}}{2\sum_{j}{\tilde{p}}_{j}{\tilde{q}}_{j}}-1$$

To gain insight into this formula, note that if ${\tilde{p}}_{ik1}$ = 1/2 for all loci, Equation 29 simplifies to 2*ψ* − 1, where *ψ* is the fraction of homozygous loci. In the context of an IBS model, homozygosity is an appropriate state quantity for measuring the inbreeding of an individual. The overall inbreeding coefficient *f* can be written as an average over individuals or over loci

(30) $$\sum_{i=1}^{n}n^{-1}\phi_{i}=\sum_{k=1}^{\tilde{m}}\beta_{k}f_{k}=f$$

where the weights *β_k_* are given in Equation 12.

Because the allele content at each locus is centered by the population mean, our realized relationship matrix is positive semidefinite but not strictly positive definite (there is at least one zero eigenvalue). This means the breeding values follow a singular normal distribution, but this poses no problem from the perspective of mixed model theory (Searle *et al.* 1992).

### Heritability

When the genetic covariance is written as proportional to the numerator relationship matrix, the proportionality constant is the additive genetic variance in the outbred base population. Because the IBS-relationship matrix uses the current population as the “base,” one might expect its proportionality constant, $\sigma^{2}=2\sigma_{u}^{2}\langle {\tilde{p}}_{k}{\tilde{q}}_{k}\rangle$ (Equation 11), to equal the genetic variance of the current population, but this is not true for inbred lines. As originally shown by Fisher (1941) [see also Kempthorne (1957) and Lynch and Walsh (1998)], the additive genetic variance for a single locus with no dominance is $\sigma_{A}^{2}=2\sigma_{u}^{2}pq\left(1+f\right)$. Compared with the coefficient of the relationship matrix, the additive genetic variance is larger by a factor of (1+*f*).

This fact has implications for estimating heritability in the narrow sense. If the additive genetic values in the mixed model are breeding values (*i.e.* twice the mean progeny value; see *Appendix 1*), heritability can be defined using parent-offspring regression as

(31) $$h^{2}=\frac{{\text{cov}}_{i}\left[a_{i},y_{i}\right]}{{\text{var}}_{i}\left[y_{i}\right]}=\frac{a\prime \left(y-\mu 1\right)}{{\left\|y-\mu 1\right\|}^{2}}$$

Replacing the breeding values (and phenotypic mean *μ*) in Equation 31 with their predicted values provides an immediate estimator for heritability. By taking the expected value of Equation 31, heritability can be related to the variance components of the mixed model (Equation 26). In File S1, we show that for large populations

(32) $$E\left[h^{2}\right]\approx \frac{\sigma^{2}\left(1+f\right)}{\sigma^{2}\left(1+f\right)+\sigma_{e}^{2}}$$

Equation 32 can also be used to estimate *h*^2^ by replacing the variance components with their ML or REML estimates.

### Shrinkage

Yang *et al.* (2010) proposed using the identity matrix as a low-dimensional target when shrinking the estimate of the relationship matrix: ${\hat{A}}^{*}=\delta I+\left(1-\delta \right)\hat{A}$. For inbred populations, this estimator is not ideal because it shrinks the off-diagonal and diagonal elements with the same intensity. By contrast, our estimator does not shrink the inbreeding coefficient.

Using both real and simulated phenotypes, we have demonstrated that shrinkage can substantially increase the accuracy of GEBVs for phenotyped individuals (or lines), but not for unphenotyped ones. Although the term “genomic selection” is typically used in the context of predicting unphenotyped individuals, it is also encompasses the selection of phenotyped individuals for mating based on GEBV, which is important in plant and animal breeding. In plant breeding, we also see potential to use the realized relationship matrix with single-replicate or unbalanced multi-environment yield trials to more accurately advance lines for variety or hybrid development, and shrinkage may be beneficial in these applications.

### Conclusion

There were two objectives in this study. The first was to formulate the realized relationship matrix based on identity-by-state at causal loci and by requiring the mean diagonal element to equal 1+*f* for the current population. For high-density markers, the optimal estimator of this relationship matrix is equivalent to the first formula of VanRaden (2008). The second objective was to explore shrinkage estimation of the relationship matrix at low marker density. In unstructured populations with more lines than markers, shrinkage estimation can increase the accuracy of GEBVs for phenotyped lines; there is no benefit without phenotypes. Particularly when phenotypes have moderate accuracy, *e.g.* from preliminary yield trials in plant breeding, shrinkage estimation has the potential to improve the selection of lines as parents or for variety development.

## Supplementary Material

Supporting Information

## Appendix 1

We prove that, for an additive trait in an arbitrary population, the formula in Equation 4 for the genetic value of line *i*

$$a_{i}=\sum_{k=1}^{\tilde{m}}\left({\tilde{X}}_{ik}-2{\tilde{p}}_{k}\right)u_{k}\equiv \sum_{k}{\tilde{W}}_{ik}u_{k}$$

equals two times the mean genetic value of its progeny under random mating, expressed relative to the population mean. In other words, the additive genetic value is the breeding value. By assuming the genetic effects *u_k_* are physiological parameters of the causal loci (rather than defined in a least-squares sense), we can write the progeny genetic value as $g_{ij}=\sum_{k}{\tilde{Z}}_{jk}u_{k}$ where ${\tilde{Z}}_{jk}$ is the allele dosage at locus *k* in progeny *j*. The mean progeny value for line *i* is thus

(33) $$\text{E}\left[g_{ij}|{\tilde{X}}_{i•}\right]=\sum_{k}\text{E}\left[{\tilde{Z}}_{jk}|{\tilde{X}}_{ik}\right]u_{k}$$

where the expectation is with respect to the random processes of gamete segregation and mating. The former contributes $\frac{1}{2}{\tilde{X}}_{ik}$ and the latter contributes ${\tilde{p}}_{k}$ for a mean progeny value of $\sum_{k}\left(\frac{1}{2}{\tilde{X}}_{ik}+{\tilde{p}}_{k}\right)u_{k}$. Subtracting the population mean $2\sum_{k}{\tilde{p}}_{k}u_{k}$ and multiplying by two produces Equation 4. Note that Equation 33 does not require the causal loci to be in linkage equilibrium (the linearity of the expectation operator does not require statistical independence).

## Appendix 2

We prove that for a single locus, the inbreeding coefficient defined by Equation 10 (written here without overscript tildes)

$$f_{k}=\frac{n^{-1}\sum_{i}\left(x_{ik1}-p_{k}\right)\left(x_{ik2}-p_{k}\right)}{p_{k}q_{k}}$$

also quantifies the deviation from Hardy-Weinberg proportions. For allele content *X_i_* ∈ {0,1,2}, the proportion of heterozygotes (*H*) is

(34) $$H=n^{-1}\sum_{i=1}^{n}X_{i}(2-X_{i})$$

Upon substituting *X_i_* = *x_i_*_1_ + *x_i_*_2_ into Equation 34, where *x_i_*_1_ and *x_i_*_2_ are binary variables denoting the two gametes in individual *i*, the result is

(35) $$\begin{array}{c} H=n^{-1}\sum_{i=1}^{n}\left(x_{i1}+x_{i2})(2-x_{i1}-x_{i2}\right)\\ =n^{-1}\sum_{i}(x_{i1}+x_{i2}-2x_{i1}x_{i2})\\ =2p-2n^{-1}\sum_{i}(x_{i1}-p+p)(x_{i2}-p+p)\\ =2p-2p^{2}-2pqf\\ =2pq(1-f) \end{array}$$

Similarly, the proportion of *X_i_* = 2 homozygotes is (omitting several steps)

(36) $$\begin{array}{c} P=\frac{1}{2}n^{-1}\sum_{i=1}^{n}X_{i}\left(X_{i}-1\right)\\ =n^{-1}\sum_{i}x_{i1}x_{i2}\\ =pqf+p^{2} \end{array}$$
