# Supplementary material for: Shrinkage Estimation of the Realized Relationship Matrix
Source: G3 (Bethesda). 2012 Nov 1;2(11):1405–13. doi: 10.1534/g3.112.004259 (PMC3484671; doi:10.1534/g3.112.004259)
Supplement: Supporting Information [file supp_2_11_1405__index.html]

Supporting Information 

# Shrinkage Estimation of the Realized Relationship Matrix

## Supporting Information for Endelman and Jannink, 2012

**Files in this Data Supplement:**

- Supporting Information - Figure S1 and S2 and File S1 (PDF, 227 KB)
- Figure S1 - Histograms of the off-diagonal realized relationship coefficients (PDF, 63 KB)
- Figure S2 - Maximizing accuracy vs. minimizing MSE for the 2+6-row barley population (see Figure 3 caption) (PDF, 68 KB)
- File S1 - Supporting text (PDF, 164 KB)
