# Supplementary material for: Shrinkage Estimation of the Realized Relationship Matrix
Source: G3 (Bethesda). 2012 Nov 1;2(11):1405–13. doi: 10.1534/g3.112.004259 (PMC3484671; doi:10.1534/g3.112.004259)
Supplement: Supporting Information [file supp_2.11.1405_004259SI.pdf]

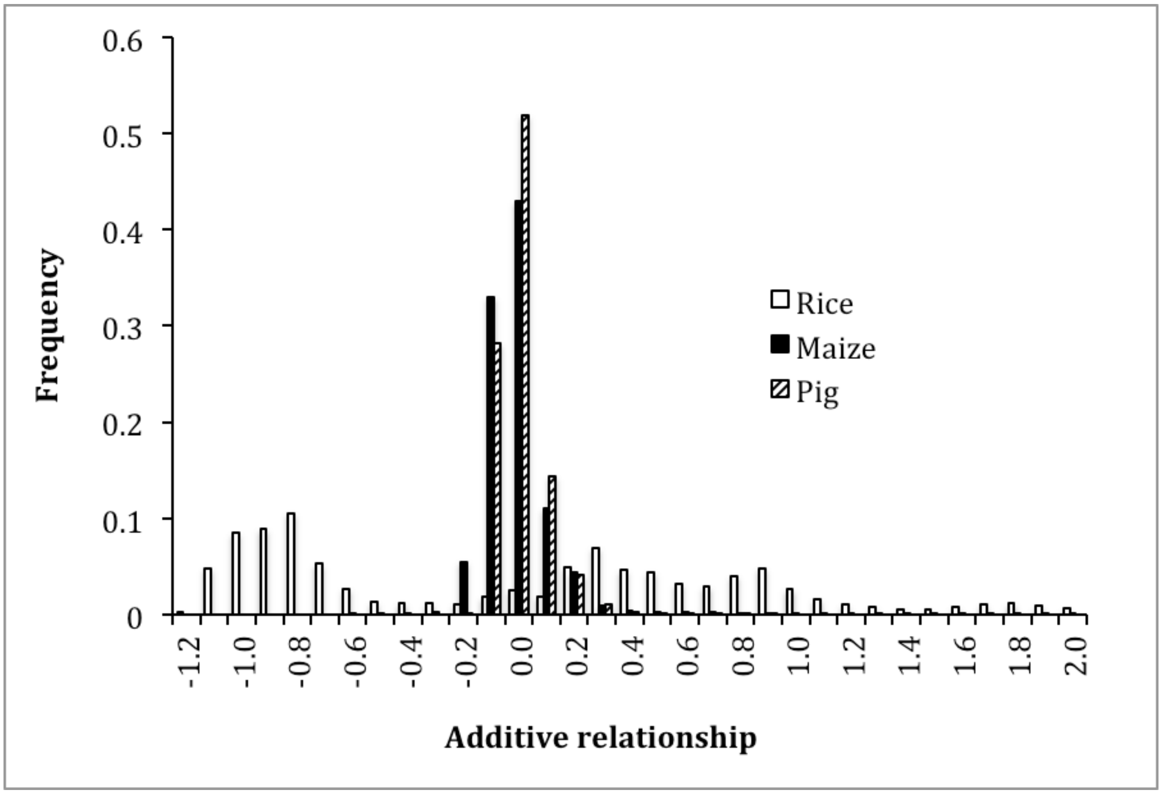

Figure S1 Histograms of the off-diagonal realized relationship coefficients.

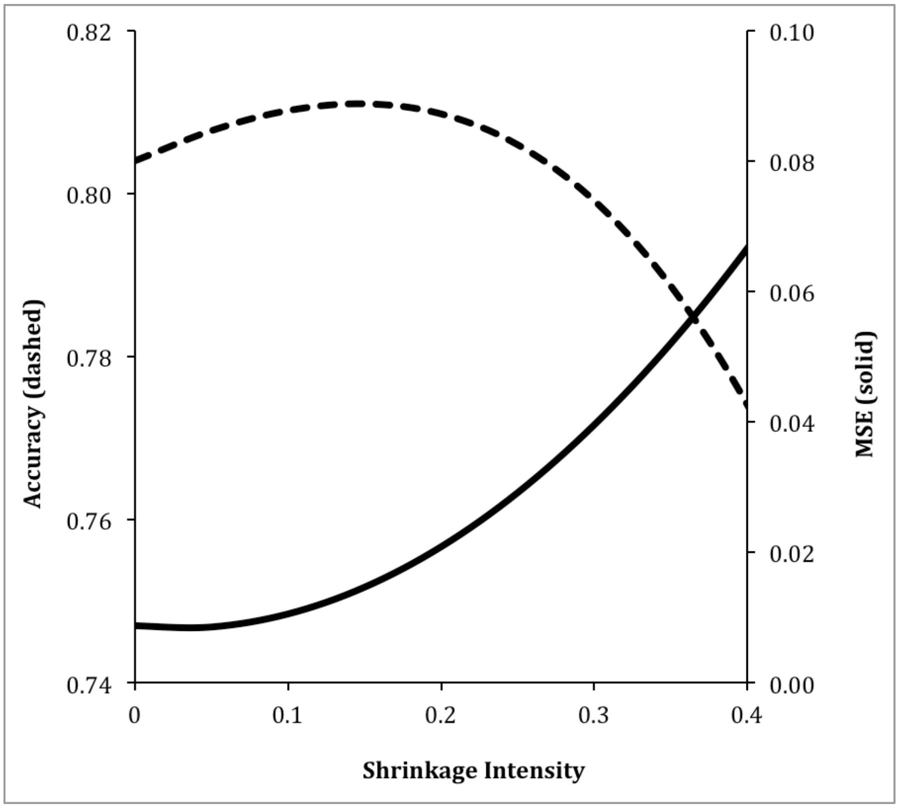

Figure S2 Maximizing accuracy vs. minimizing MSE for the 2+6-row barley population (see Figure 3 caption).

File S1

Starting from the parent-offspring regression (Equation 31):

$$h^2 = \frac{\text{cov}_i\left[a_i, y_i\right]}{\text{var}_i\left[y_i\right]} = \frac{\mathbf{a}'\left(\mathbf{y} - \mu\mathbf{1}\right)}{\left\|\mathbf{y} - \mu\mathbf{1}\right\|^2}$$

we derive an expression for heritability in terms of the variance components of the mixed model $\mathbf{y} = \mu\mathbf{1} + \mathbf{a} + \boldsymbol{\varepsilon}$, where $\mathbf{a} \sim N\left(0, \mathbf{G}\right)$, $\mathbf{G} = \mathbf{A}\sigma^2$, and $\boldsymbol{\varepsilon} \sim N\left(0, \mathbf{I}\sigma_e^2\right)$. The numerator (*N*) and denominator (*D*) in Equation 31 are univariate random variables that can be written as quadratic forms:

$$\begin{aligned} N &\equiv \mathbf{a}'\left(\mathbf{y} - \mu\mathbf{1}\right) = \mathbf{z}'\mathbf{Q}_N\mathbf{z} \\ D &\equiv \left(\mathbf{y} - \mu\mathbf{1}\right)'\left(\mathbf{y} - \mu\mathbf{1}\right) = \mathbf{z}'\mathbf{Q}_D\mathbf{z} \end{aligned} \tag{S1}$$

where the stacked vector **z** and matrices $\mathbf{Q}_N$, $\mathbf{Q}_D$ are

$$\begin{aligned} \mathbf{z} &= \begin{bmatrix} \mathbf{a} \\ \mathbf{y} - \mu\mathbf{1} \end{bmatrix} \sim N\left(\begin{bmatrix} \mathbf{0} \\ \mathbf{0} \end{bmatrix}, \begin{bmatrix} \mathbf{G} & \mathbf{G} \\ \mathbf{G} & \mathbf{G} + \sigma_e^2\mathbf{I} \end{bmatrix}\right) \\ \mathbf{Q}_N &= \tfrac{1}{2}\begin{bmatrix} \mathbf{0} & \mathbf{I} \\ \mathbf{I} & \mathbf{0} \end{bmatrix} \\ \mathbf{Q}_D &= \begin{bmatrix} \mathbf{0} & \mathbf{0} \\ \mathbf{0} & \mathbf{I} \end{bmatrix} \end{aligned} \tag{S2}$$

Since $\text{E}\left[\mathbf{z}'\mathbf{Q}\mathbf{z}\right] = \text{tr}\left(\mathbf{Q}\mathbf{V}\right)$ for $\mathbf{z} \sim N(\mathbf{0}, \mathbf{V})$ (Searle 1971), it follows that

$$\begin{aligned} \text{E}\left[N\right] &= \text{E}\left[\mathbf{z}'\mathbf{Q}_N\mathbf{z}\right] = \tfrac{1}{2}\text{tr}\left(\begin{bmatrix} \mathbf{0} & \mathbf{I} \\ \mathbf{I} & \mathbf{0} \end{bmatrix}\begin{bmatrix} \mathbf{G} & \mathbf{G} \\ \mathbf{G} & \mathbf{G} + \sigma_e^2\mathbf{I} \end{bmatrix}\right) = \text{tr}\left(\mathbf{G}\right) \\ \text{E}\left[D\right] &= \text{E}\left[\mathbf{z}'\mathbf{Q}_D\mathbf{z}\right] = \text{tr}\left(\begin{bmatrix} \mathbf{0} & \mathbf{0} \\ \mathbf{0} & \mathbf{I} \end{bmatrix}\begin{bmatrix} \mathbf{G} & \mathbf{G} \\ \mathbf{G} & \mathbf{G} + \sigma_e^2\mathbf{I} \end{bmatrix}\right) = \text{tr}\left(\mathbf{G}\right) + n\sigma_e^2 \end{aligned} \tag{S3}$$

Since the trace of **G** equals $n(1+f)\sigma^2$, an approximate formula for heritability is

$$\mathrm{E}\left[h^2\right]=\mathrm{E}\left[\frac{N}{D}\right]\approx\frac{\mathrm{E}[N]}{\mathrm{E}[D]}=\frac{(1+f)\sigma^2}{(1+f)\sigma^2+\sigma_e^2} \tag{S4}$$

Equation S4 is only approximate because the expectation of the ratio of two random variables does not equal the ratio of expectations. By Taylor series expansion a more accurate formula is (Rice 2006)

$$\mathrm{E}\left[\frac{N}{D}\right]=\frac{\mathrm{E}[N]}{\mathrm{E}[D]}+\frac{\left(\mathrm{var}[D]\mathrm{E}[N]-\mathrm{cov}[N,D]\mathrm{E}[D]\right)}{\mathrm{E}^3[D]}+\cdots \tag{S5}$$

To investigate the magnitude of the correction term, note that for $\mathbf{z}\sim N(\mathbf{0},\mathbf{V})$,

$$\begin{aligned}\mathrm{var}\left[\mathbf{z}'\mathbf{Q}_D\mathbf{z}\right]&=2\,\mathrm{tr}\left(\left(\mathbf{Q}_D\mathbf{V}\right)^2\right)\\ \mathrm{cov}\left[\mathbf{z}'\mathbf{Q}_D\mathbf{z},\mathbf{z}'\mathbf{Q}_N\mathbf{z}\right]&=2\,\mathrm{tr}\left(\mathbf{Q}_D\mathbf{V}\mathbf{Q}_N\mathbf{V}\right)\end{aligned} \tag{S6}$$

(Searle 1971). Upon substituting S2 into S6 and simplifying, the result is

$$\begin{aligned}\mathrm{var}[D]&=2n\left[\sigma^4 n\left\langle A_{ij}^2\right\rangle+2\sigma^2\sigma_e^2(1+f)+\sigma_e^4\right]\\ \mathrm{cov}[N,D]&=2n\left[\sigma^4 n\left\langle A_{ij}^2\right\rangle+\sigma^2\sigma_e^2(1+f)\right]\end{aligned} \tag{S7}$$

where

$$\left\langle A_{ij}^2\right\rangle=n^{-2}\sum_{i=1}^{n}\sum_{j=1}^{n}A_{ij}^2 \tag{S8}$$

is the mean-squared element of **A**. From Equation S7 the correction is

$$\frac{\left(\mathrm{var}[D]\mathrm{E}[N]-\mathrm{cov}[N,D]\mathrm{E}[D]\right)}{\mathrm{E}^3[D]}=\frac{2\sigma^4\sigma_e^2\left[(1+f)^2-n\left\langle A_{ij}^2\right\rangle\right]}{n\left[\sigma_e^2+\sigma^2(1+f)\right]^3}\overset{n\to\infty}{\approx}\frac{-2\left\langle A_{ij}^2\right\rangle\sigma^4\sigma_e^2}{\left[\sigma_e^2+\sigma^2(1+f)\right]^3} \tag{S9}$$

where the last expression holds for large populations.

Using Equation 13, the mean-squared realized relationship is

$$\begin{aligned}\left\langle A_{ij}^2 \right\rangle &= \left(2\left\langle \tilde{p}_l \tilde{q}_l \right\rangle\right)^{-2} n^{-2} \sum_{i=1}^{n} \sum_{j=1}^{n} \sum_{k=1}^{\tilde{m}} \sum_{k'=1}^{\tilde{m}} \left(\tilde{X}_{ik'} - 2\tilde{p}_{k'}\right)\left(\tilde{X}_{ik} - 2\tilde{p}_k\right)\left(\tilde{X}_{jk'} - 2\tilde{p}_{k'}\right)\left(\tilde{X}_{jk} - 2\tilde{p}_k\right) \\ &= \left(2\left\langle \tilde{p}_l \tilde{q}_l \right\rangle\right)^{-2} \tilde{m}^{-2} \sum_k \sum_{k'} r_{kk'}^2 \tilde{p}_k \tilde{q}_k \tilde{p}_{k'} \tilde{q}_{k'}\end{aligned} \tag{S10}$$

where

$$r_{kk'} = \left(\tilde{p}_k \tilde{q}_k \tilde{p}_{k'} \tilde{q}_{k'}\right)^{-1/2} n^{-1} \sum_{i=1}^{n} \left(\tilde{X}_{ik} - 2\tilde{p}_k\right)\left(\tilde{X}_{ik'} - 2\tilde{p}_{k'}\right) \tag{S11}$$

is the correlation between loci. If the correlation length between causal loci is small compared to the size of the genome, then $\left\langle A_{ij}^2 \right\rangle$ will also be small. For example, if the causal loci are in linkage equilibrium, then

$$\left\langle A_{ij}^2 \right\rangle = \frac{\left\langle \tilde{p}_k^2 \tilde{q}_k^2 \right\rangle}{4\tilde{m}\left\langle \tilde{p}_l \tilde{q}_l \right\rangle^2} \tag{S12}$$

More generally we can write $\left\langle A_{ij}^2 \right\rangle = c/\tilde{m}$ for some finite constant *c*, which upon substitution in Equation S9 shows the correction term tends to zero under the infinitesimal model ($\tilde{m} \to \infty$).

Literature Cited

Rice, J., 2006 *Mathematical Statistics and Data Analysis,* 3rd ed. Duxbury, Pacific Grove.

Searle, S. R., 1971 *Linear Models*. John Wiley & Sons, New York.
