# Supplementary figures and images for: Shrinkage Estimation of the Realized Relationship Matrix
Source: G3 (Bethesda). 2012 Nov 1;2(11):1405–13. doi: 10.1534/g3.112.004259 (PMC3484671; doi:10.1534/g3.112.004259)

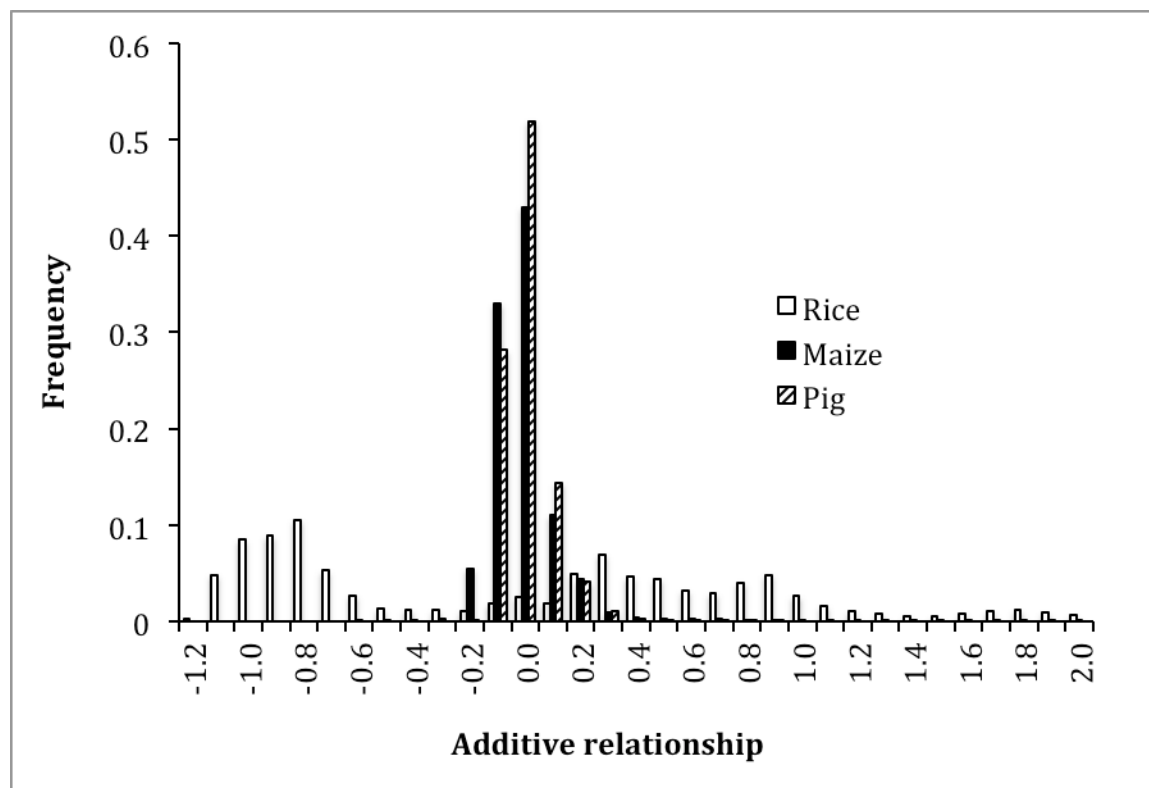

**Figure S1** Histograms of the off-diagonal realized relationship coefficients.

Supplement: Supporting Information [file supp_2.11.1405_FigureS1.pdf]
