# Supplementary material for: Shrinkage Estimation of the Realized Relationship Matrix
Source: G3 (Bethesda). 2012 Nov 1;2(11):1405–13. doi: 10.1534/g3.112.004259 (PMC3484671; doi:10.1534/g3.112.004259)
Supplement: Supporting Information [file supp_2.11.1405_FigureS2.pdf]

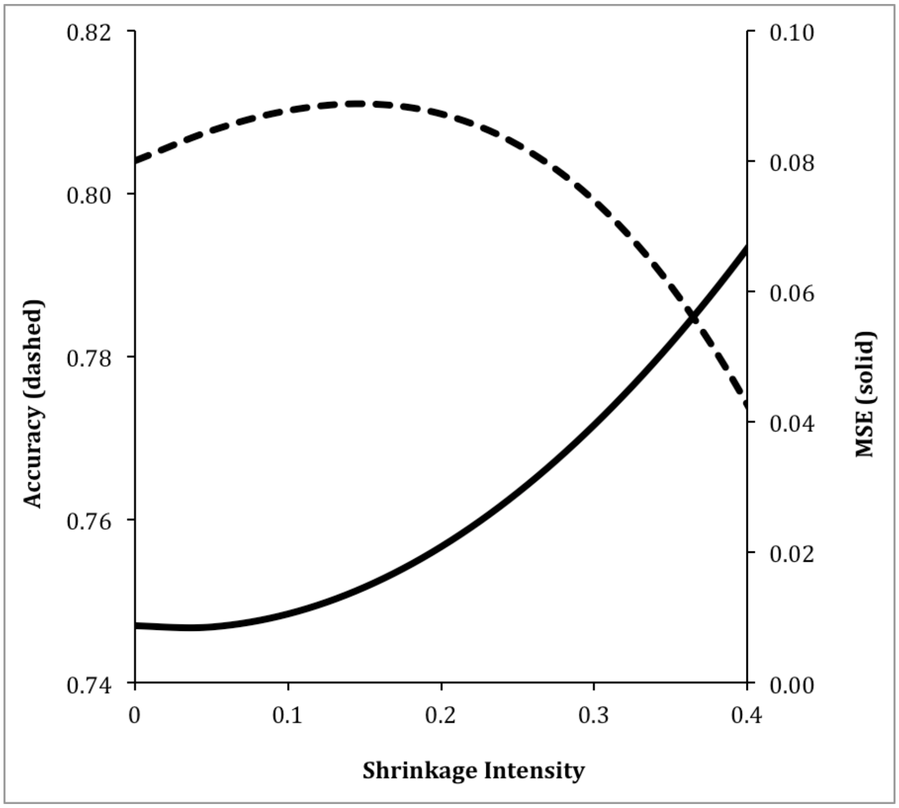

Figure S2 Maximizing accuracy vs. minimizing MSE for the 2+6-row barley population (see Figure 3 caption).
